# Medially positioned plate in first metatarsophalangeal joint arthrodesis

**DOI:** 10.1371/journal.pone.0260572

**Published:** 2021-12-01

**Authors:** Wojciech Witkowski, Leszek Kuik, Magdalena Rucka, Karol Daszkiewicz, Angela Andrzejewska, Piotr Łuczkiewicz

**Affiliations:** 1 Department of Mechanics of Materials and Structures, Faculty of Civil and Environmental Engineering, Gdańsk University of Technology, Gdańsk, Poland; 2 II Clinic of Orthopaedics and Kinetic Organ Traumatology, Medical University of Gdansk, Gdańsk, Poland; University of Vigo, SPAIN

## Abstract

**Objective:**

The purpose of this study was to biomechanically compare the stability of first metatarsophalangeal (MTP1) joint arthrodesis with dorsally and medially positioned plates.

**Methods:**

A physical model of the MTP1 joint consists of printed synthetic bones, a titanium locking plate and screws. In the experiments, samples with dorsally and medially positioned plates were subjected to loading of ground load character in a universal testing machine. Force-displacement relations and relative displacements of bones were recorded. The obtained results were used to validate the corresponding finite element models of the MTP1 joint. Nonlinear finite element simulations of the toe-off phase of gait were performed to determine the deformation and stress state in the MTP1 joint for two positions of the plate.

**Results:**

In numerical simulations, the maximum displacement in the dorsal direction was noticed at the tip of the distal phalanx and was equal to 19.6 mm for the dorsal plate and 9.63 mm for the medial plate for a resultant force of 150 N. Lower relative bone displacements and smaller plastic deformation in the plate were observed in the model with the medial plate. Stress values were also smaller in the medially positioned plate and locking screws compared to fixation with the dorsal plate.

**Conclusions:**

A medially positioned locking plate provides better stability of the MTP1 joint than a dorsally positioned plate due to greater vertical bending stiffness of the medial plate. Smaller relative bone displacements observed in fixation with the medial plate may be beneficial for the bone healing process. Moreover, lower stress values may decrease the risk of complications associated with hardware failure.

## Introduction

Arthrodesis of the first metatarsophalangeal (MTP1) joint is a commonly performed procedure in the treatment of end-stage arthritis [1–3]. A good outcome following this procedure depends on maintenance of the bone position during the postoperative period and a fixation strong enough to achieve bone union. The techniques that have been described for MTP1 joint arthrodesis include the use of an external fixator, Kirschner wires, staples, dorsal precontoured locking and nonlocking plates or a combination of these methods [4–8]. Although MTP1 joint arthrodesis is commonly described as a safe and effective procedure, the incidence of surgical revision after this procedure is not insignificant. The incidence of nonunion ranges from 5.4% to 10%, and hardware removal rates comprise 8.5% of cases [9, 10]. The major causes of failures are related to the hardware. Inadequate arthrodesis fixation not only causes nonunion but can also result in an average change in the dorsiflexion angle during the postoperative period [11]. For this reason, the current literature (see, e.g., [12, 13]) focuses on techniques of internal fixation strong enough to allow early weight bearing without loss of the initial bone stability. Harris et al. [14] found that the most stable fixation method is a dorsal plate with a lag screw. Recently, Kuik and Łuczkiewicz [15] described for the first time the use of a medial approach with a medially positioned plate for MTP1 joint arthrodesis. The authors concluded that a medially positioned locking plate and the use of a medial approach seem to be valuable options for the MTP1 arthrodesis procedure. However, a biomechanical comparison of dorsally and medially positioned plates has not been performed so far.

The aim of this study was to biomechanically compare constructs with a dorsally positioned plate and a medially positioned plate in MTP1 joint stabilization. The analysis of MTP1 joint stiffness was performed on the physical and numerical models.

## Materials and methods

### Geometry

A three-dimensional model of the first metatarsophalangeal joint was developed from the freely accessible STL geometry of the human foot [16]. Unfortunately, the model does not contain detailed information about the donor. Thus, the STL geometry was imported to Hypermesh (Altair Engineering, Troy, Michigan, USA) and scaled to match standard dimensions of the proximal phalanx described by Munuera et al. [17]. The articulating MTP1 joint surfaces were cut to a congruent shape in the model, which corresponds to using a concave proximal reamer and a distal convex reamer during operation. Next, the bones were cut to allow dorsal and medial placement of the plate. Such a prepared geometry was used for the 3D printing of joint physical models and finite element analysis (FEA).

### Physical models

The joint physical models were shaped by a fused filament fabrication (FFF) procedure with the use of an Ultimaker 3 Extended 3D printer (Ultimaker B.V., Utrecht, Netherlands). The solid object, described by a polygon mesh in STL format, was processed in Ultimaker Cura, a parametric 3D print design software. The joint physical models were printed with an infill density of 100% with white Ultimaker polylactide (PLA). Ultimaker polyvinyl alcohol (PVA) was used to build the supports. Then, the supports were washed in distilled water for 12 hours. The washing time of the supports had no effect on the mechanical behavior of the FFF polylactide. The applied 3D printing parameters for polylactide were as follows: nozzle temperature– 220°C; building platform temperature– 60°C; nozzle diameter– 0.4 mm; filament diameter– 2.85 mm; single layer thickness– 0.1 mm; internal infill pattern–lines (+45°/-45°). The mechanical parameters of fused filament fabricated polylactide were determined on dog-bone shaped specimens in a uniaxial tensile test. The density of polylactide was determined using a cube with a side length of 20 mm. The measured values were: Young’s modulus *E* = 2.35 GPa; Poisson’s ratio *v* = 0.36; density *ρ* = 1045 kg/m^3^; see also Table 1.

**Table 1:** Mechanical elastic properties.

Material	Young’s modulus [GPa]	Poisson’s ratio [–]	Density [kg/m^3^]
Titanium	110	0.37	4400
Steel	210	0.3	7800
PLA	2.35	0.36	1045
Damaged PLA	0.47	0.36	1045

**Fig 1:**
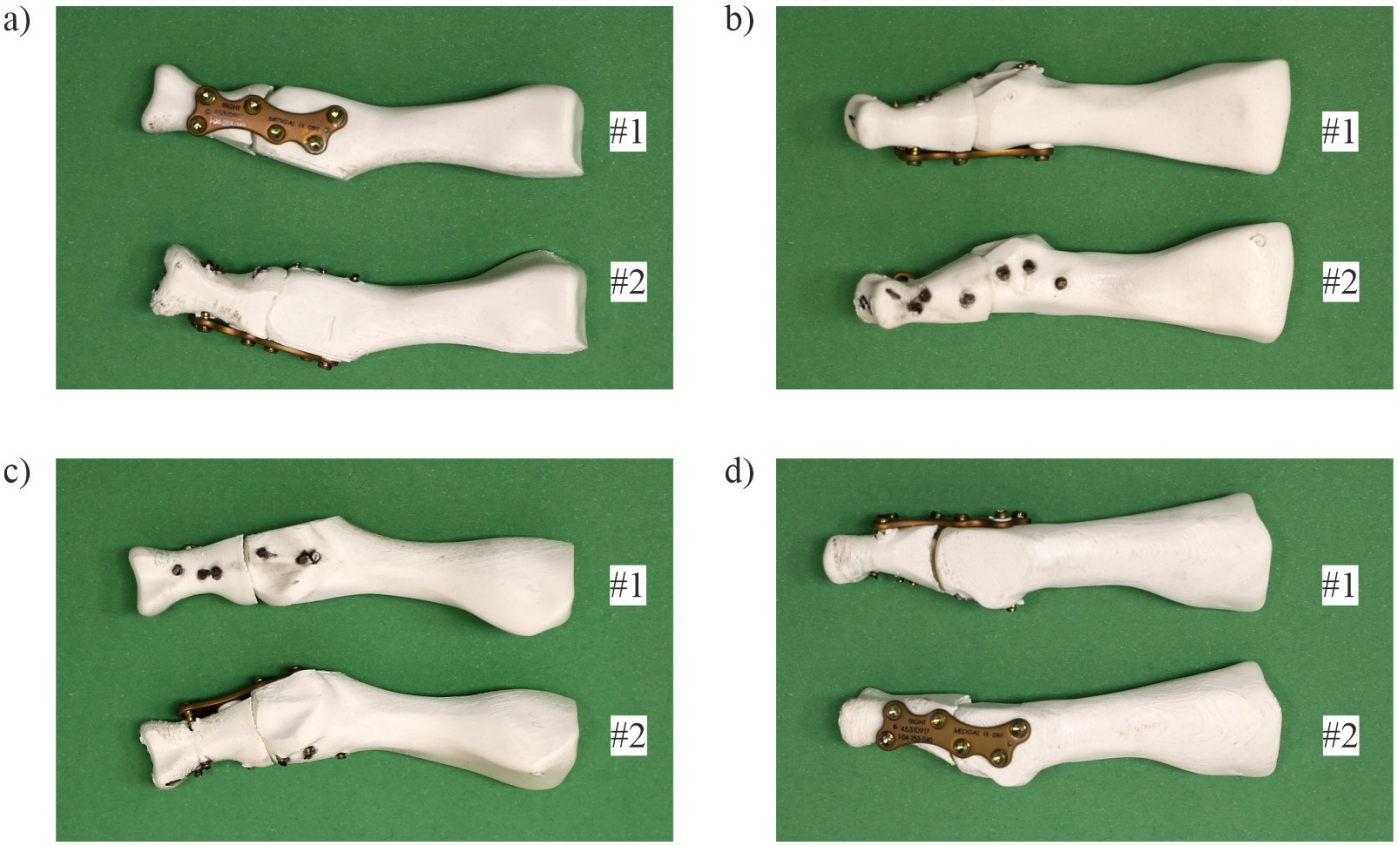
Tested samples (#1 –joint with a dorsally positioned plate and #2 –joint with a medially positioned plate): a) dorsal view; b) lateral view; c) plantar view; d) medial view.

A titanium locking plate held by six screws was used in the MTP1 joint fusion. Two samples (#1 and #2) were prepared for further experimental analysis. The first (sample #1) was a joint with a dorsally positioned plate, while the second (sample #2) was a joint with a medially positioned plate. The prepared joints are presented in Fig 1.

### Experiment

**Fig 2:**
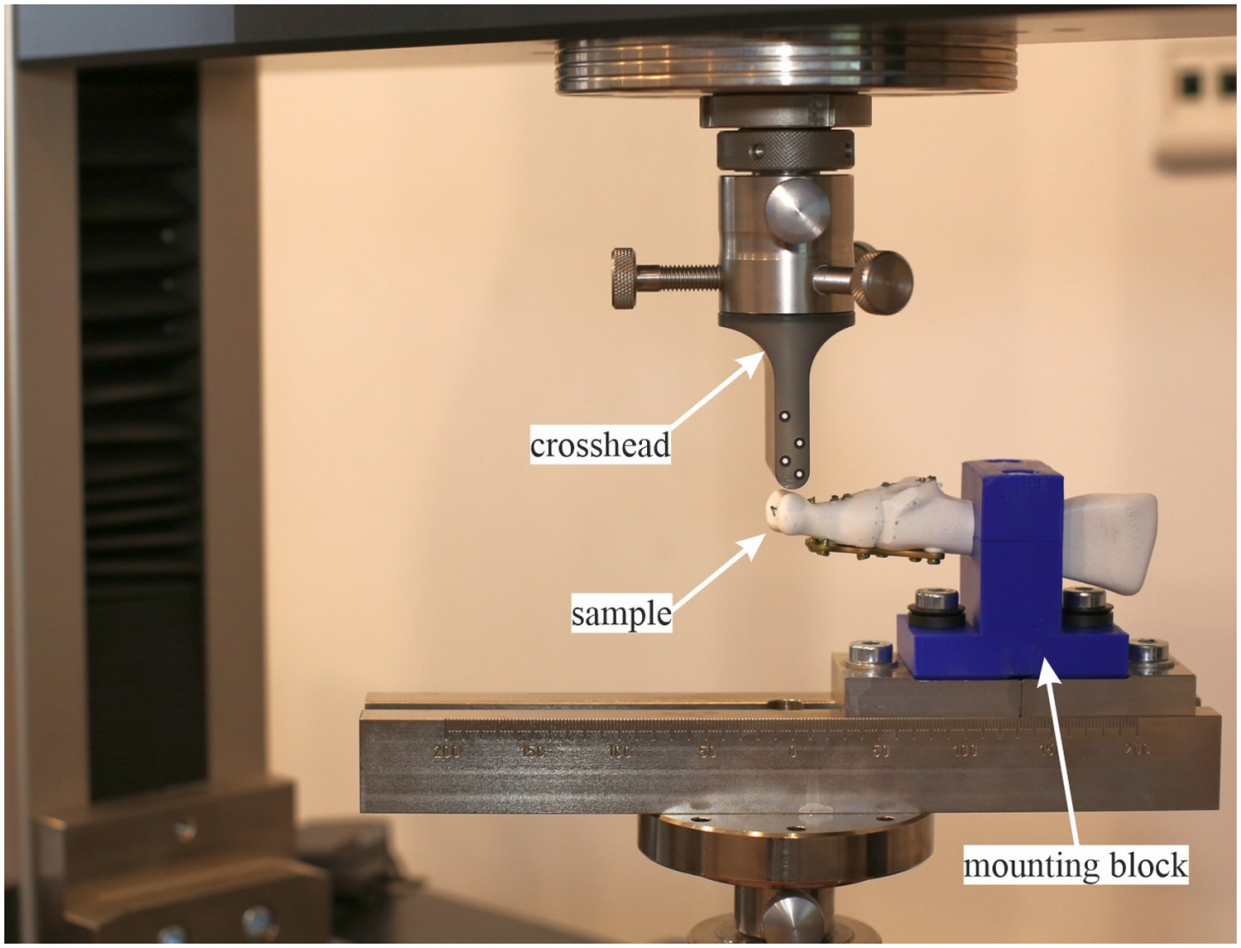
Experimental setup for the bending test.

The bending tests on samples #1 and #2 were performed in a Zwick/Roell Z10 universal testing machine (ZwickRoell GmbH & Co. KG, Ulm, Germany). The experimental setup is illustrated in Fig 2. The MTP1 joints were fixed in a specially designed and fabricated block. The homemade mounting block was fitted to the bone geometry and 3D printed with the parameters described above. Both elements of the mounting block were connected with stainless steel screws and then fixed to the testing machine.

In the beginning of the test, a preload of 0.2 N was applied, after which the process of bending was carried out with a constant vertical displacement increment of 1 mm/min.

**Fig 3:**
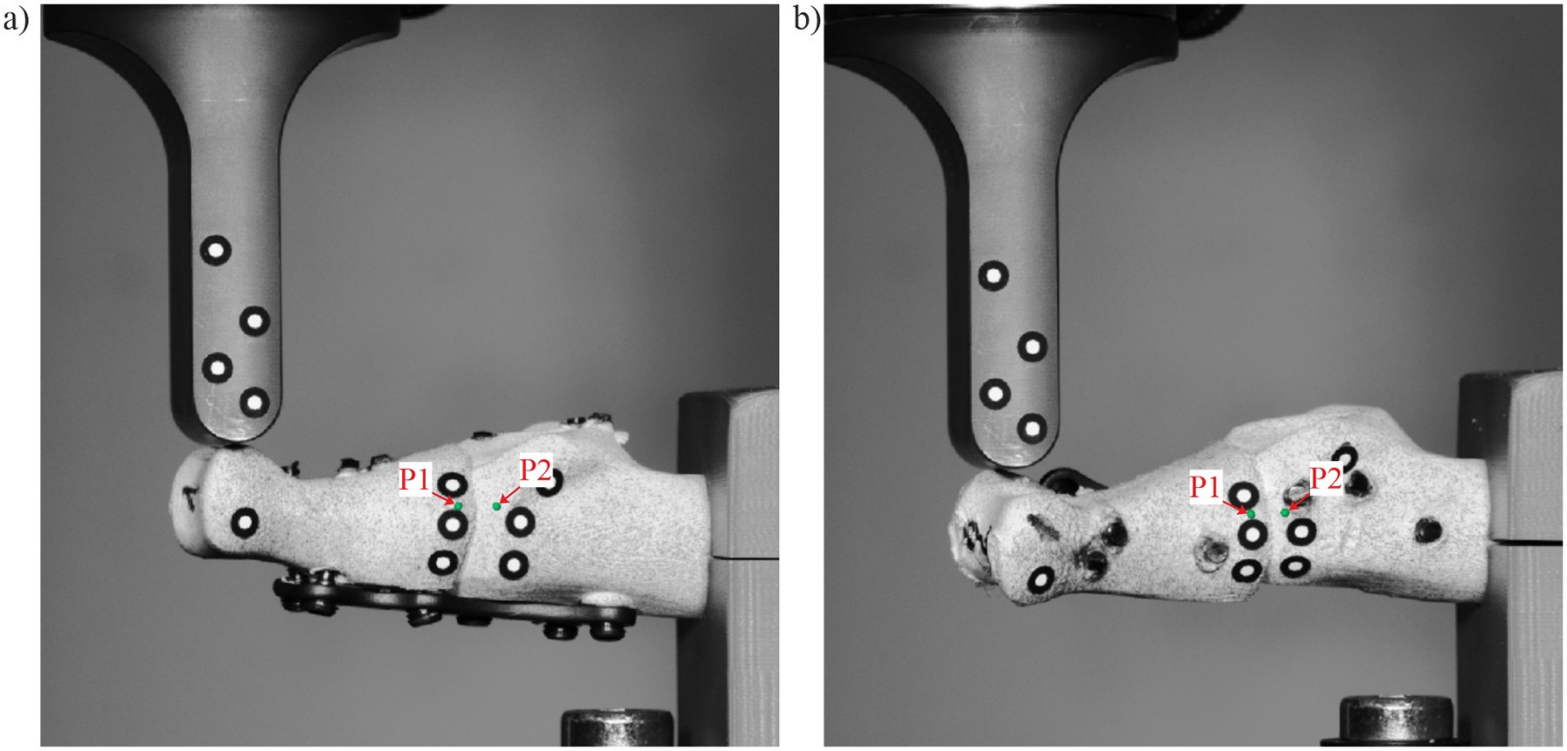
Lateral view from the DIC camera: a) specimen #1; b) specimen #2.

During the bending test, the digital image correlation (DIC) technique was applied to observe the mechanical behavior of the joints. Fig 3 shows views from cameras at the beginning of the tests. Photographs of the lateral surface of the samples, covered with a stochastic pattern, were taken every 1 s using an ARAMIS Adjustable 12M system (GOM GmbH, Braunschweig, Germany). Then, the images were processed by ARAMIS Professional software (GOM GmbH, Braunschweig, Germany). For further validation, two control points (points P1 and P2, marked in green color in Fig 3) were selected on the stochastic pattern. Dorsal and axial relative displacements between these points were registered.

### Finite element method models

**Fig 4:**
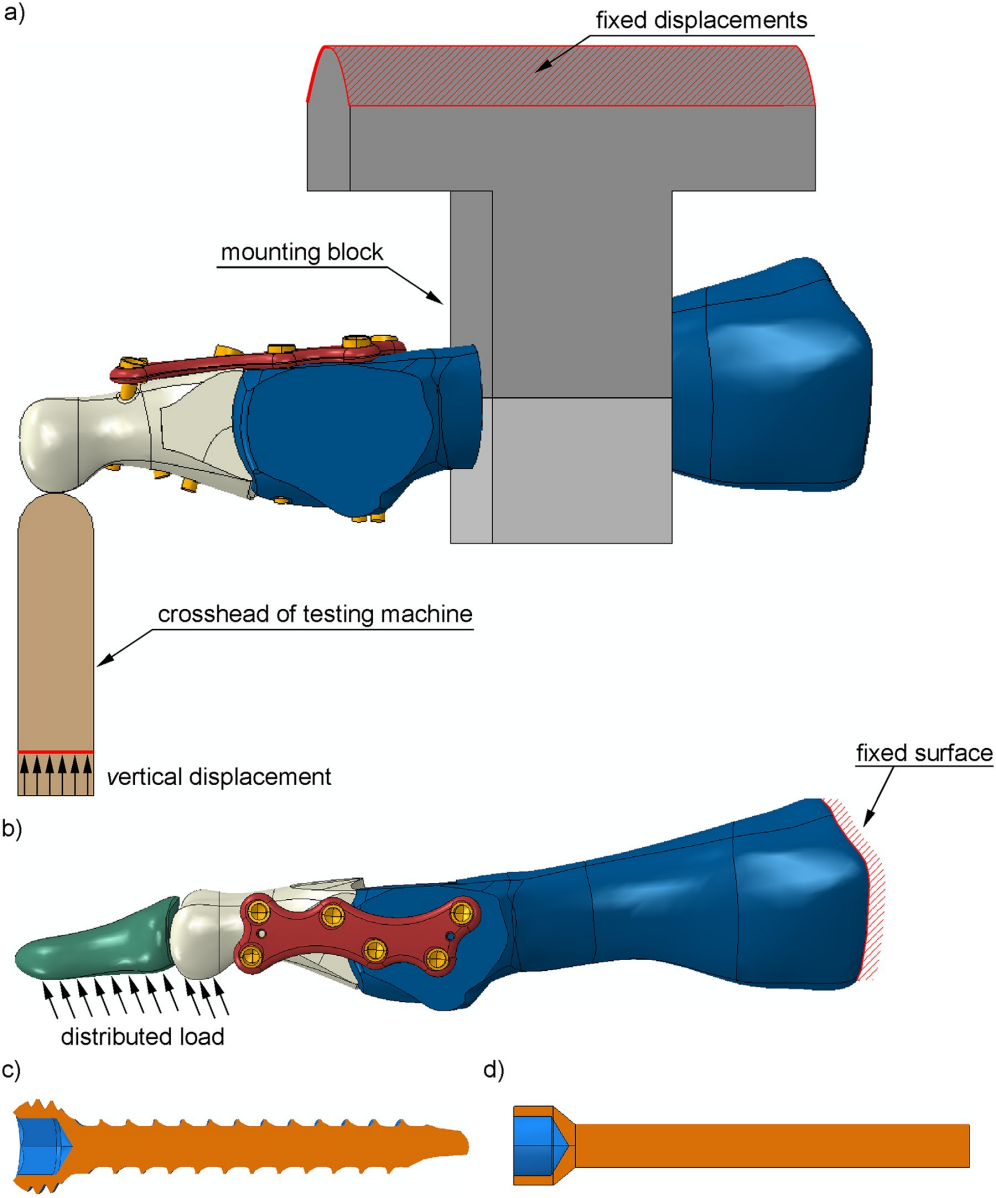
Geometry of the numerical models and boundary conditions used in a) simulation of the experiment for the dorsal plate and b) simulation of the toe-off phase of gait for the medial plate. Cross section through c) the original screw and d) simplified screw used in the models.

A general view of the numerical model is presented in Fig 4. A simplified geometry of the plate and screws with no threads (Fig 4d) was created based on the dimensions of the original plate and screws (Fig 4c) used in the experiment. Moreover, the bones, plate and screws were positioned in Hypermesh to reflect their positions in the experiment. The prepared geometry of the MTP1 joint for the dorsal and medial positions of the plate was exported to the Abaqus v. 6.14 (Dassault Systemes Simulia Corp., Providence, USA) finite element method (FEM) system. The composite mounting block and the crosshead of the testing machine with a half-cylinder end were included in simulations of the experiment (Fig 4a). A distal phalanx was considered only in simulations of the toe-off phase of gait (Fig 4b).

#### Material parameters

**Fig 5:**
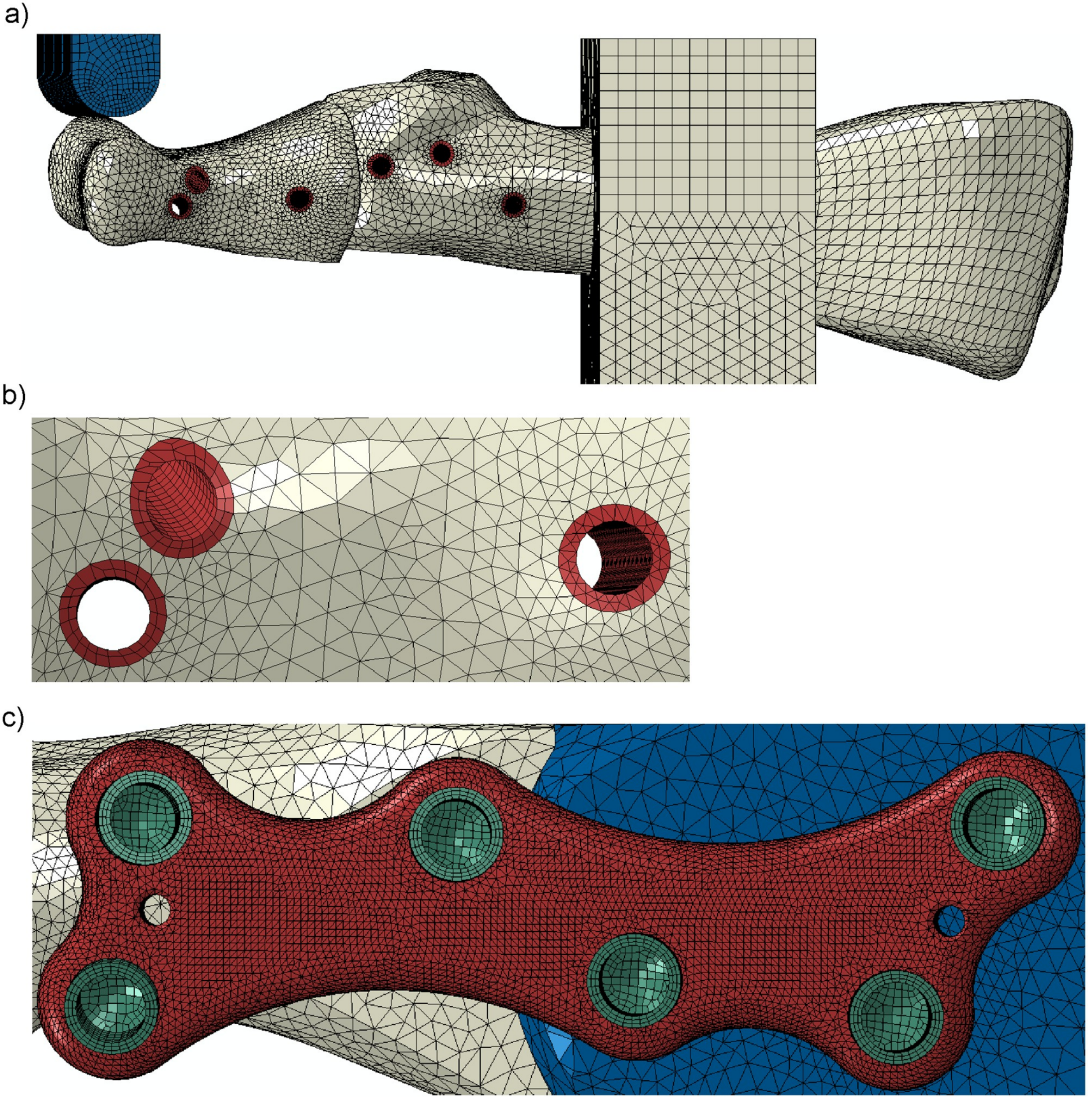
Finite element mesh of the MTP1 joint model: a) lateral view; b) the vicinity of the screws in the proximal phalanx; c) titanium plate and screws.

The elastic properties of the materials used in the model of the MTP1 joint are given in Table 1. The Johnson–Cook constitutive equation with the following values of material parameters [18]: *A* = 997.9 MPa, *B* = 653.1 MPa, *n* = 0.45, and *m* = 0.7 was applied to describe the plasticity with isotropic hardening for the titanium plate and screws. A linear elastic, perfectly plastic material model was assumed for the bones and mounting block printed from the PLA filament. The yield stress *σ*_y_ = 49.5 MPa for the PLA material was taken from the manufacturer’s data [19]. Consideration of insertion-related bone damage was necessary for the validation of the finite element (FE) model in a study [20]. Screw insertion in the trabecular bone causes the most bone damage within a 0.3 mm radial distance from the screw [21]. For screws with a small thread size of 0.3 mm used in experiments, further bone damage was observed up to a radial distance of 0.6 mm from the screw [21]. Taking this into account, the radial thickness of the bone damage regions around the screws was assumed to be 0.45 mm for the composite bones in the numerical models (see Fig 5b, the regions marked in red). A reduced Young’s modulus (see Table 1) and a lower yield stress *σ*_yd_ = 15.5 MPa corresponding to 3.3% elongation at yield [19] were assigned to the finite elements in these regions.

#### Mesh

The plate, bones, screws, crosshead and mounting block were modeled using 3-dimensional solid elements (Fig 5a). First-order hexahedral finite elements (C3D8) were applied in discretization of the screws, crosshead, top part of the mounting block (Fig 5a) and some regions of damaged bone around the screws (Fig 5b). Tetrahedral elements were used to discretize other parts of the model for which the use of hexahedral elements was not possible due to the irregular shape of their geometry. The bones and bottom part of the mounting block were discretized by first-order tetrahedral finite elements (C3D4) for which smaller contact convergence problems were observed. The titanium plate was meshed using 10-node tetrahedral elements (C3D10) with quadratic shape functions (Fig 5c) to obtain accurate contour maps of effective stress in the plate and screws. The discretization was refined in the regions of stress concentration. Solid elements of approximately 0.3 mm in size were used in the FE mesh of the titanium plate, screws and bones around the screws. The average size of the elements in the phalanges and the crosshead of the testing machine were assumed to be 1 mm. Finite elements of approximate sizes of 1.5 mm and 2 mm were applied in discretization of the metatarsal bone and mounting block, respectively. The whole model used in the simulation of the experiment consisted of 461,995 and 465,949 finite elements for the dorsally positioned plate and medially positioned plate, respectively. The discretization was positively verified by convergence analysis.

#### Interactions

A hard contact approach with enabled separation after contact was used to model the interaction between the proximal phalanx and metatarsal bone, between the screws and bones, between the screws and titanium plate and between the proximal phalanx and cylindrical surface of the crosshead. A friction coefficient μ = 0.2 was assumed for the contact surfaces of the proximal phalanx. We used the lower value of the coefficient than 0.28 determined for the white filament in an experimental study [22], taking into account that the crosshead of the testing machine and the titanium plate had lower roughness than the steel used in the study [22]. Moreover, the parametric analysis showed good agreement between experimental and numerical results for the value of 0.2. A high coefficient of friction μ = 2.0 was applied for the contact surfaces of the screws to reduce slip due to the presence of a thread in the original screws.

### Simulation of the experiment

The metatarsal bone was rigidly connected to the mounting block using the *TIE constraint in the simulations of the experiment. All translational degrees of freedom were constrained at the top part of the mounting block (see Fig 4a). The bottom surface of the crosshead was fixed with the exception of its dorsal (vertical) translation. A quasi-static type of implicit nonlinear dynamic analysis was chosen to minimize dynamic effects in the FE computations.

### Simulation of the toe-off phase

Once the FEM model was validated against the experimental results, the behavior of the MTP1 joint for each position of the plate was simulated in the toe-off phase of gait. This phase was chosen due to the maximal value of the ground reaction force (GRF) on the hallux [23]. The rigid constraint was applied between the distal and proximal phalanges in the simulations. Boundary conditions were defined in the FE model by fixing all degrees of freedom on the tarsometatarsal articulation of the metatarsal bone (Fig 4b). A uniformly distributed following load was applied to the plantar surface of the distal phalanx and anterior part of the proximal phalanx to simulate the GRF (see Fig 4b). A ratio of 0.4 between the anterior and vertical components of the GRF was assumed based on the values in Table 2 from Ref. [23] for the toe-off phase. Nonlinear dynamic analysis was performed, accounting for large displacements, plasticity and contact effects.

**Table 2:** Comparison of maximal relative displacements [mm] between bone surfaces at the MTP1 joint for a resultant force of 150 N. The relative bone translations were also computed in the center of the proximal phalanx surface.

	Direction	Dorsal plate	Medial plate
Maximal	lateral	0.7	0.65
	dorsal	1.76	1.36
	axial	4.39	1.02
Center	lateral	0.12	-0.13
	dorsal	1.7	1.01
	axial	2.17	0.45

## Results

### Validation

**Fig 6:**
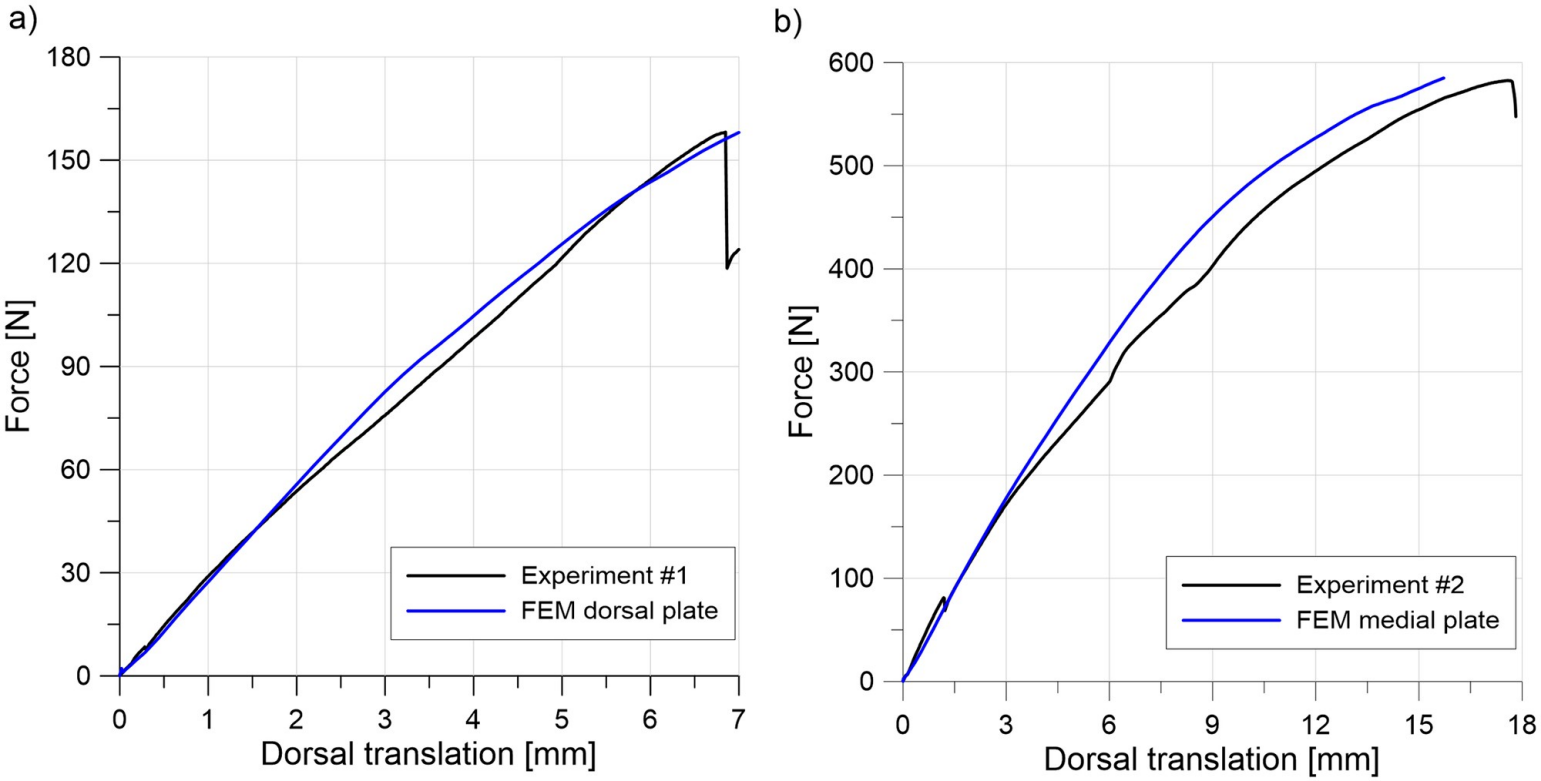
Validation of the model force-displacement curves for a) a dorsally positioned plate and b) a medially positioned plate.

**Fig 7:**
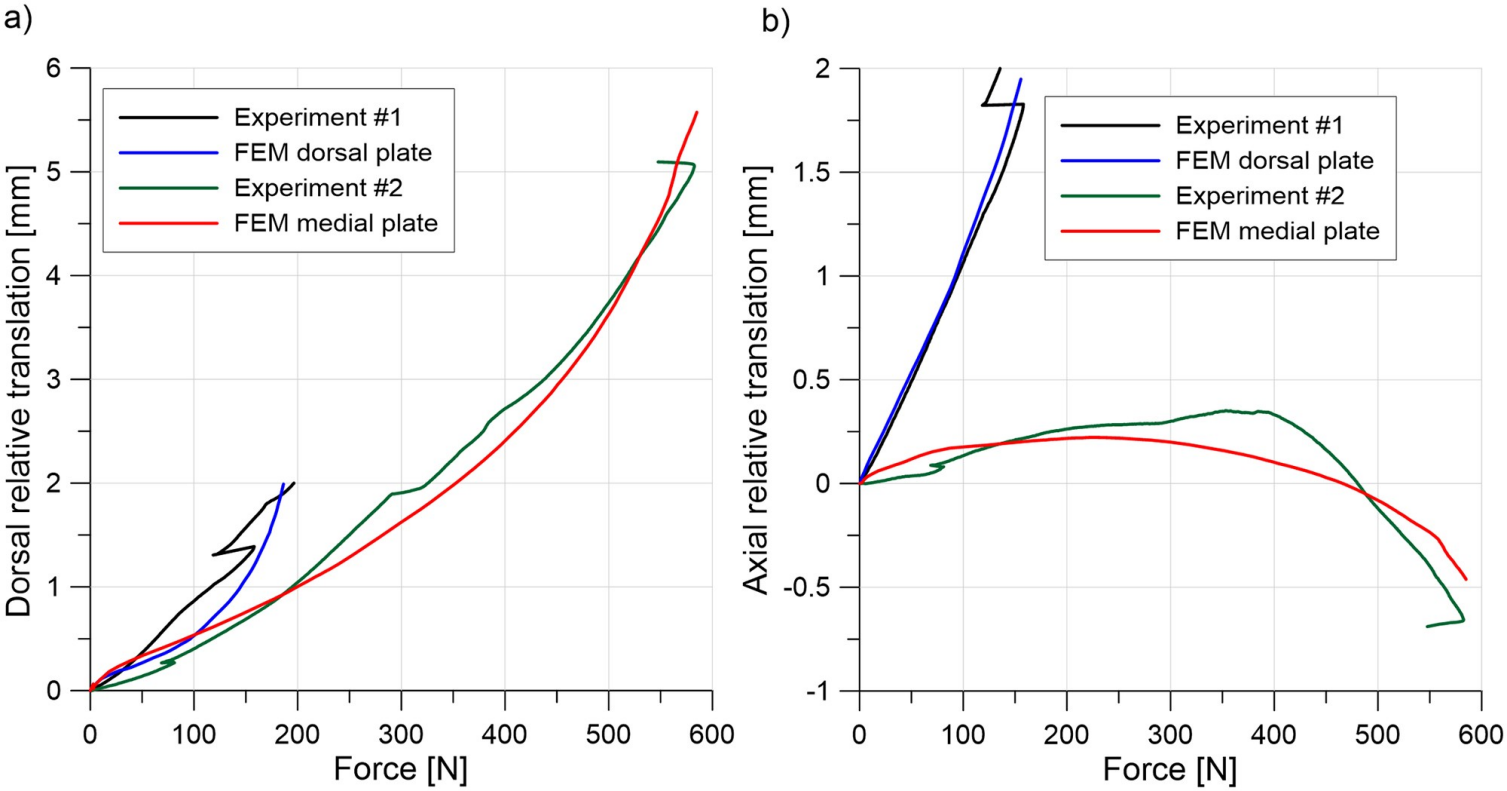
Validation of dorsal (a) and axial (b) translations of point P1 relative to point P2 for dorsally and medially positioned plates.

For validation, Fig 6 depicts the relation between the vertical force and deflection of the crosshead of the testing machine for dorsally and medially positioned plates. The FE model predicts the experimental results well. In the experiment, the movement between the two screws closest to the crosshead and dorsally positioned plate caused a decrease in force (see Fig 6a); therefore, further results for greater deflections were omitted. Failure of bone was observed for the medially positioned plate. Translations of point P1 relative to point P2 (see Fig 3) are presented in Fig 7 to validate the relative displacements between bones in the MTP1 joint. Considerably greater relative displacements were obtained for dorsally positioned plate, especially in the experiment. Good agreement between the numerical and experimental curves was observed.

### Toe-off phase

**Fig 8:**
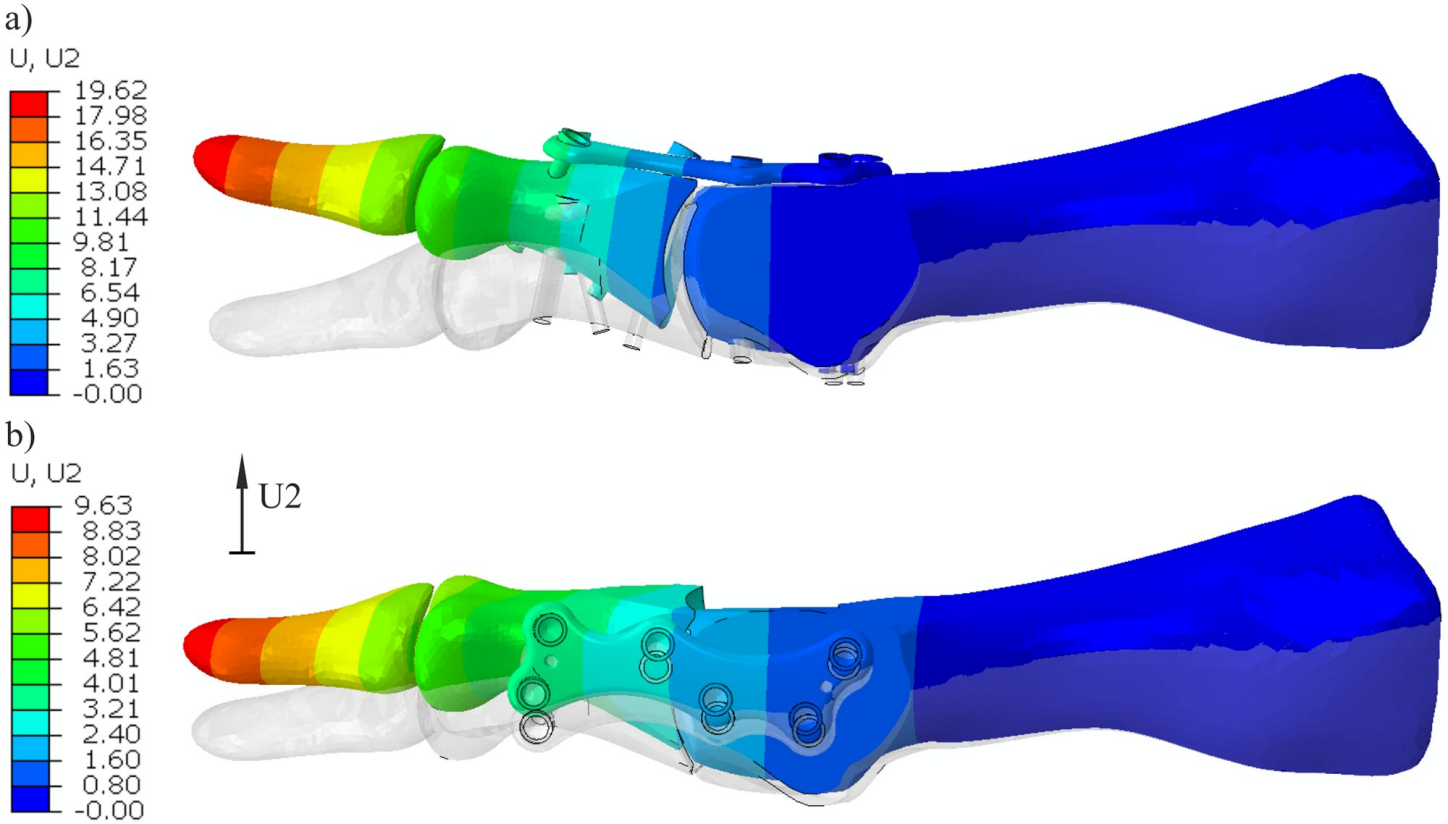
Dorsal displacements [mm] in the FEM analysis of models with a) a dorsal plate and b) a medial plate.

Fig 8 shows a comparison of dorsal displacements from FEM computations for both plate positions in the simulated toe-off phase of gait. The initial position of the joint is shown in gray. All discussed values are shown for a resultant load of 150 N. Maximum displacement in the dorsal direction is noticed at the tip of the distal phalanx and equals 19.6 mm for the model with the dorsal plate and 9.63 for the model with the medial plate.

**Fig 9:**
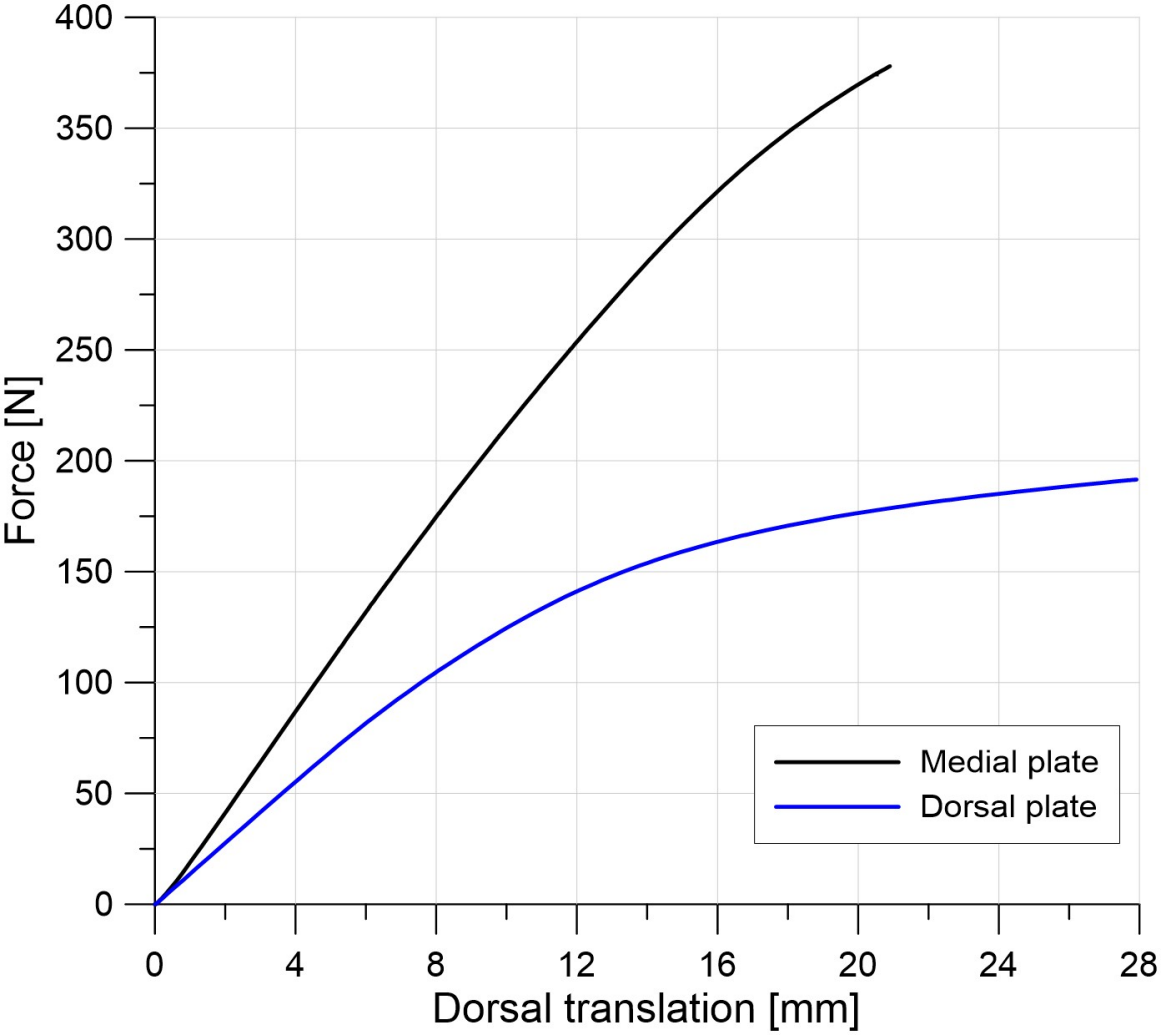
The relationship between the force applied to the FE model and the dorsal translation of point P3 at the distal phalanx (see Fig 4b) for both plate positions.

The force-displacement curves show that fixation with the medial plate gives greater stiffness of MTP1 joint fusion in the toe-off phase than fixation with the dorsal plate (see Fig 9). As in the experiment, a substantially greater maximal load was obtained in the model with the medial plate, despite the computations being interrupted due to convergence issues. We verified that the equilibrium equations were satisfied during the simulation and too large correction of displacements on the contact surface between bone and screw caused the termination of the analysis.

**Fig 10:**
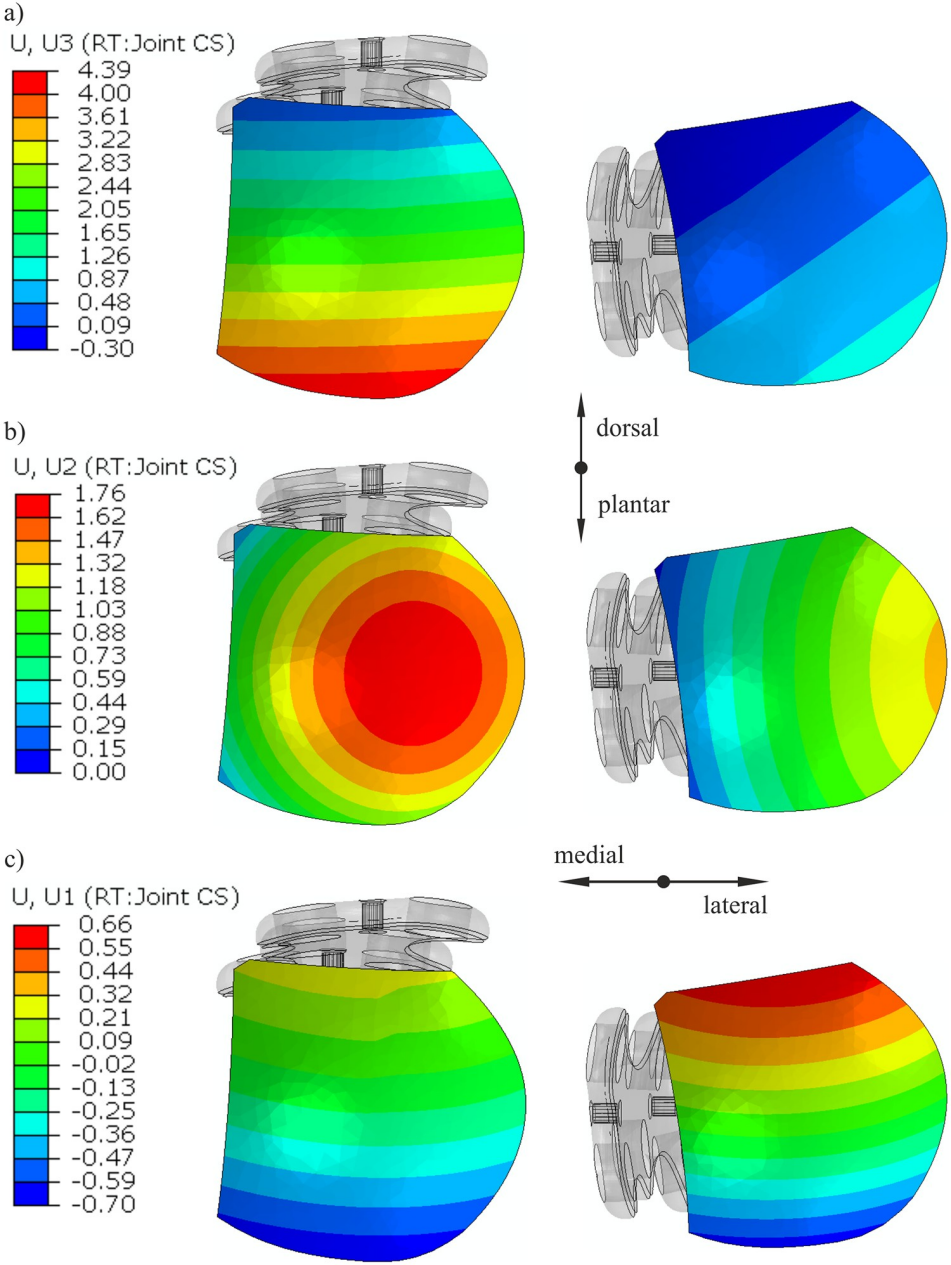
Displacements [mm] of the proximal phalanx surface relative to the metatarsal bone surface for a resultant load of 150 N: a) axial translation; b) dorsal translation; c) medial (+)/ lateral (-) translation.

Fig 10 shows contour maps of displacements of the proximal phalanx surface relative to the metatarsal bone surface inside the MTP1 joint for dorsal plate fixation (left column) and medial plate fixation (right column). The largest relative displacements were observed in the axial direction on the plantar edge of the phalanx surface with the dorsal plate. Approximately four times smaller maximal axial relative translations were obtained for the medial plate construct (see Table 2). In addition to maximal relative displacements, values at the center of the proximal phalanx surface are given in Table 2 because they are close to the mean values.

The greatest component of relative displacements for the model with a medially positioned plate was the dorsal component. However, even larger dorsal relative displacements were noticed approximately at the center of the surface with a dorsally positioned plate (Fig 10b). The relative translations in the lateral direction are comparable for both plate positions (Table 2). Only the maximal relative medial displacement is greater for the model with the medial plate (0.66 mm) than for the model with the dorsal plate (~0.3 mm) (see Fig 10c).

**Fig 11:**
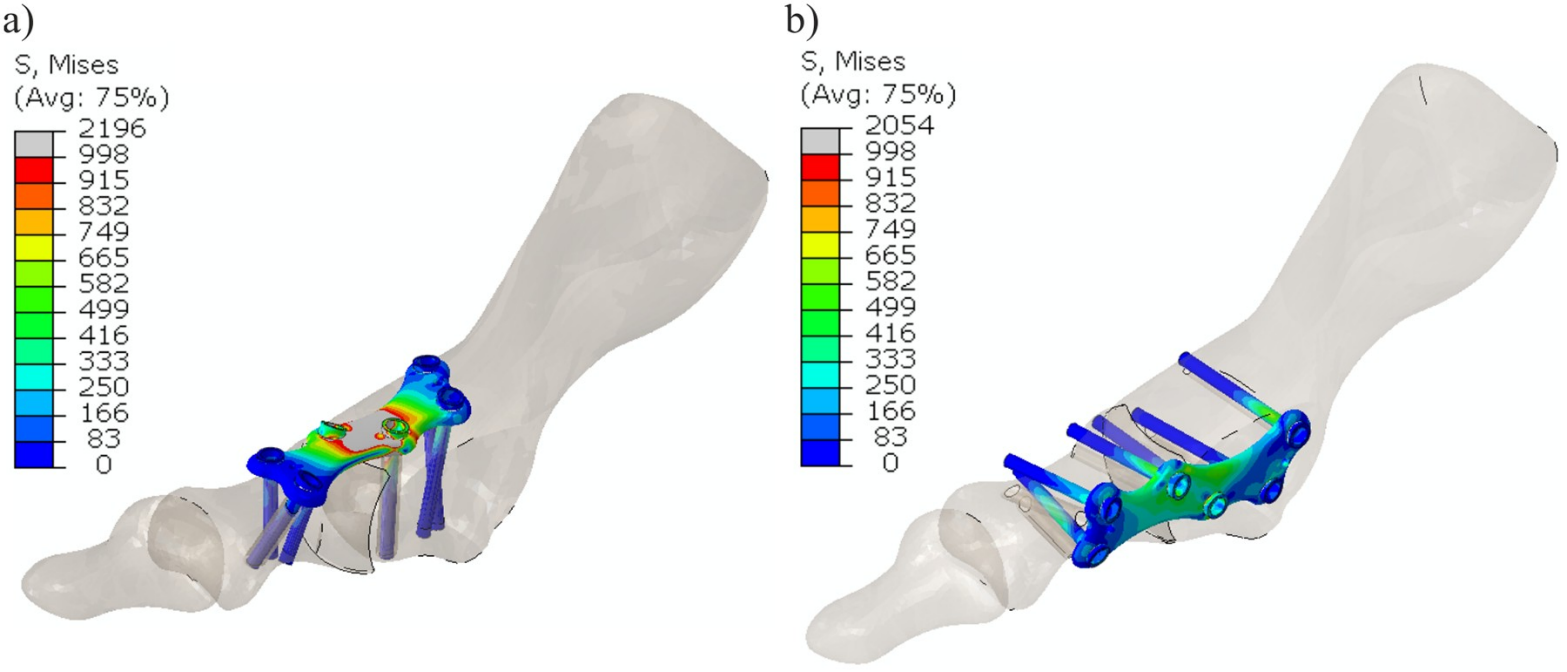
Contour maps of the effective stress [MPa] for models with a) a dorsal plate and b) a medial plate.

**Fig 12:**
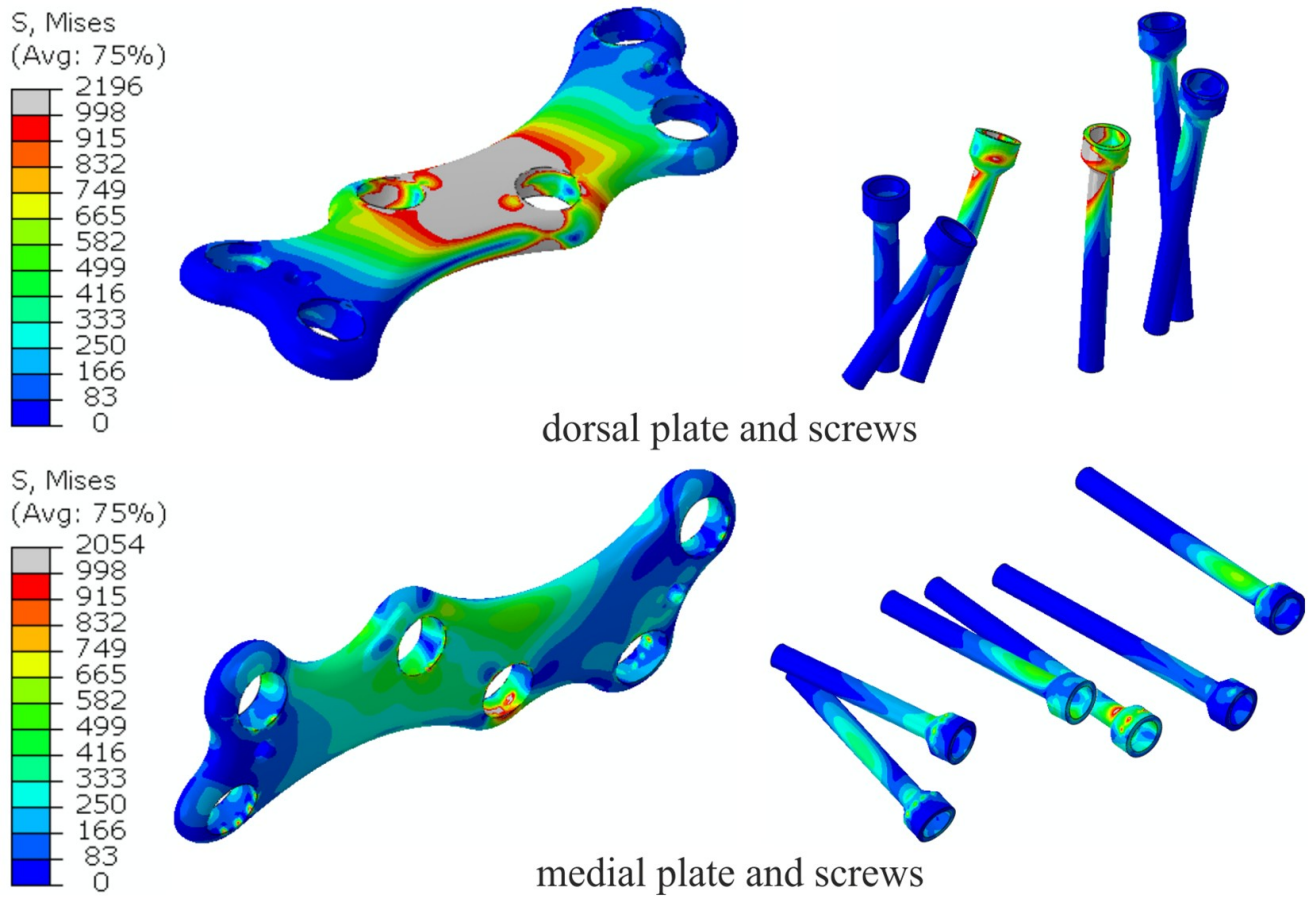
Detailed view of the effective stress [MPa] distribution in the plates and screws for both plate positions.

Contour maps of the Huber-Mises-Hencky stress (effective stress) are presented in Fig 11. Fig 12 shows a detailed view of the effective stress distribution in the plates and locking screws. Gray denotes areas where the stress is larger than the yield stress of the hardware material. In dorsal placement, large portions of the plate at the MTP1 joint are in a plastic state for a resultant force of 150 N. In the medially positioned plate and screws, the effective stress amounts to ~660 MPa with only local peak values, and in the model with the dorsal plate, plasticity was also observed in some regions of the two middle screws closest to the MTP1 joint.

## Discussion

The medial incision approach to the MTP1 joint comes with a range of benefits, such as superior cosmetic appearance, good maintenance of perfusion of the metatarsal head, and safe distance from the dorsomedial cutaneous nerve of the hallux [24]. For the first time, Kuik and Łuczkiewicz [15] described the medial approach with a medially positioned plate for MTP1 joint arthrodesis. In our study, we chose to use FEA to determine whether a medially positioned plate has better biomechanical properties than a dorsally positioned plate. This method has proven to be a very useful tool for creating a complex model of bone stabilization and has been widely applied in the research of orthopedic biomechanics, e.g., [25]. Since the essential step in this method is the validation of FEM models, we confirmed that our numerical results are consistent with experimental results for the physical models.

The stability of arthrodesis fixation plays a crucial role in revascularization and the bone healing process [26]. Our analyses showed that the medial plate gives better stability of the MTP1 joint in the toe-off phase. Lienau et al. revealed that larger bone interfragmentary movement was associated with a reduced blood supply, lower bone quality and lower stiffness in the healed bone [27]. In molecular analyses, the same authors demonstrated that upregulation of genes was important for vascularization in the healing area [28]. In some studies [12, 29], a threshold value of 2 mm for the maximum acceptable relative movement between bones was used as the failure criterion. The greater relative displacements were interpreted as nonunion of the MTP1 joint fusion. Fig 8a shows a large gap on the plantar side of the joint with the dorsal plate. Locally, this gap yields large relative axial translations between the joint surfaces (see Fig 10a). Specifically, the maximal value of 4.4 mm exceeded the threshold value of 2 mm. The dorsal component of the relative displacement shows that a large portion of the proximal phalanx surface is moving relative to the first metatarsal bone with the dorsal plate. The lateral-medial relative translations have the smallest values in comparison with the remaining components. The displacements between bones are restrained when a medial plate is used. The maximal relative translation of 1.4 mm in the model with the medial plate was lower than 2 mm for a resultant force of 150 N (Table 2). Moreover, a stiffer fixation is advantageous for smaller gaps between bones because the tissue strain increases with decreasing gap size [30].

Recent studies [31, 32] have suggested that immediate full weight-bearing after MTP1 joint arthrodesis may be acceptable and beneficial. A subhallucal load may be 25% of the body weight at the toe-off phase [33], which gives an upper estimate of 150–250 N of load in the MTP1 joint. The maximal force of 192 N obtained for the numerical model with the dorsally positioned plate (Fig 9) is consistent with the approximate value of 210 N reported for the dorsal plate and synthetic bones in the study of Harris et al. [14]. Similar failure loads were obtained for the composite and cadaveric bones in the study [12]. Figs 11 and 12 demonstrate the fact that medial placement of the plate and screws is beneficial in terms of the stress state. Specifically, smaller stresses were observed in the medial plate than in the dorsally positioned plate due to the greater bending stiffness of the medial plate in the dorsal direction. The limit load capacity is almost exhausted for the force of 150 N in the model with the dorsal plate because large portions of the plate and screws undergo stresses greater than the yield point. This may result in fatigue failure of the plate material when taking into account the effect of repetitive loading on the construct. The level of maximal effective stresses observed for the medial plate fusion construct (Fig 12) leaves a safety margin for the overall bearing capacity. Considerably greater maximal load and lower stress values may allow immediate weight-bearing or reduce the non-weight bearing period in MTP1 joint arthrodesis with the medial plate. However, further research is required in this area, which considers, for example, the effect of repetitive loading and statistical analysis of the data [34].

Our study has some limitations.

- The influence of soft tissues was omitted. Moreover, muscle forces acting on the MTP1 joint at the tendon insertion sites were not taken into account in the simulation of the toe-off phase. Future simulations are planned on the more complex model of the MTP1 joint.
- One type of hardware was studied. Different geometries of the locking plate will be analyzed in future research.
- The results obtained for synthetic bones may not reflect the values for real bones. However, the use of the same synthetic bones for both plate positions allowed us to eliminate the influence of heterogeneity of cadaveric samples on the results. Moreover, the study concentrated on the plate and screws, and the stiffness of the composite bones was close to the equivalent stiffness of real bones.
- The failure mechanism was not replicated in the FE model, because an elastic, perfectly plastic material model was assumed for bone. However, the load capacity determined in the FE model is close to the limit load from the experiment. Modelling real bone fracture is planned in future research.

## Conclusions

Smaller relative translations between bones show that a medially positioned plate provides better stabilization of MTP1 joint arthrodesis than a dorsally positioned plate due to greater vertical bending stiffness of the medial plate. Moreover, lower stress values observed for the medial plate fusion construct would decrease the risk of complications associated with hardware failure.

## Supporting information

S1 Dataset Displacements and forces determined for a dorsally positioned plate. (CSV)

S2 Dataset Displacements and forces determined for a medially positioned plate. (CSV)
